# Characterizing anhedonia: A systematic review of neuroimaging across the subtypes of reward processing deficits in depression

**DOI:** 10.3758/s13415-020-00804-6

**Published:** 2020-05-29

**Authors:** Alessandra Borsini, Amelia St John Wallis, Patricia Zunszain, Carmine Maria Pariante, Matthew J. Kempton

**Affiliations:** 1 Section of Stress, Psychiatry and Immunology & Perinatal Psychiatry, King’s College London, Psychology & Neuroscience, Department of Psychological Medicine, Institute of Psychiatry, Maurice Wohl Clinical Neuroscience Institute, G.33.67, Ground Floor, Denmark Hill, London, UK; 2 King’s College London, Psychology & Neuroscience, Centre for Affective Disorders, Institute of Psychiatry, London, UK; 3 King’s College London, Psychology & Neuroscience, Department of Psychosis Studies & Department of Neuroimaging, Institute of Psychiatry, London, UK

**Keywords:** Anhedonia, Depression, Neuroimaging, fMRI, Reward processing

## Abstract

Anhedonia is a key symptom of major depressive disorder (MDD) and comprises behavioural deficits in three reward processing subtypes: reward liking, reward wanting, and reward learning. However, neuroimaging findings regarding the neural abnormalities underpinning these deficits are complex. We have conducted a systematic review to update, reframe and summarize neuroimaging findings across the three subtypes of anhedonia in MDD. Using PubMed, The Cochrane Library, PsycINFO, and Web of Science databases, we identified 59 fMRI studies comparing participants with current or remitted MDD with controls, using reward processing tasks. For reward liking and wanting, striatal hypoactivation was observed, alongside hypoactivation and hyperactivation across frontal regions. For reward learning, blunted frontostriatal sensitivity to positive feedback was observed. These findings highlight the importance of studying anhedonia not only as a clinical manifestation but also as a neurobiological mechanism underlying depressive disorder and other broader psychiatric conditions.

Major depressive disorder (MDD) is both common, with a lifetime prevalence of 16.6% in the USA (Kessler, Petukhova, Sampson, Zaslavsky, & Wittchen, 2012), and consequential, being the second leading contributor to global years lived with disability (YLDs) worldwide (Ferrari et al., 2013). Anhedonia is one of two key symptoms required for a diagnosis of MDD in the *Diagnostic and Statistical Manual of Mental Disorders, Fifth Edition* (DSM-5; American Psychiatric Association, 2013), and is defined as ‘markedly diminished interest or pleasure in all, or almost all, activities most of the day, nearly every day’ (American Psychiatric Association, 2013), and so represents a deficit in reward processing. In a study examining the factor structure for DSM-IV, MDD symptoms in a sample 2,615 army recruits, the best fit for the data indicated that MDD consisted of both a somatic and nonsomatic component (Elhai et al., 2012), and anhedonia had the second highest factor weighting (Beta = 0.76) for the nonsomatic component (after depressed mood), as well as the second highest factor weighting of all symptoms (Elhai et al., 2012). This suggests that anhedonia is a core feature of depression. Anhedonia is a symptom which warrants attention; indeed, reward processing deficits are associated with increased risk of new onset MDD (Rawal, Collishaw, Thapar, & Rice, 2013), anhedonia may precede illness onset, and, moreover, it can often persist past the remission of other depressive symptoms (Schrader, 1997), as do deficits on reward processing tasks (Pechtel, Dutra, Goetz, & Pizzagalli, 2013).

## Three subtypes of anhedonia

In DSM-5, anhedonia comprises deficits in hedonic experience of rewards and motivation for rewards (American Psychiatric Association, 2013). However, reviews have called for research to conceptualize anhedonia as comprising deficits across three partially separable subtypes of reward processing: reward liking, reward wanting, and reward learning (Admon & Pizzagalli, 2015; Rømer Thomsen, Whybrow, & Kringelbach, 2015; Treadway & Zald, 2011). Reward liking refers to the experience of pleasure from rewards; reward wanting refers to motivation driving individuals towards rewards; and reward learning refers to guiding behaviour based on previous rewards and punishments using prediction errors (PE), which signal differences between expected outcomes and what actually happens in order to support learning (Berridge & Robinson, 2003; Rømer Thomsen et al., 2015). These three subtypes of reward processing are understood to have partially separable neurobiological underpinnings (Berridge & Robinson, 2003; Rømer Thomsen et al., 2015), and behavioural deficits in each of the three subtypes make up anhedonia in MDD (Rømer Thomsen, 2015).

A more recent review also provides a more comprehensive model of anhedonia (Husain & Roiser, 2018); this involves self-cued or environmentally cued option generation; evaluation and selection between options; anticipation and preparation for action; motor mechanisms to initiative and sustain approach behaviour; a consummatory phase with positive or negative impact; and, finally, learning from the outcomes to optimize future decision-making (Husain & Roiser, 2018).

In this review, anhedonia will be conceptualized as comprising three reward processing subtypes (reward liking, reward wanting, reward learning) as it has been validated and used in several other studies (Admon & Pizzagalli, 2015; Rømer Thomsen et al., 2015; Treadway & Zald, 2011). However, these subtypes have been proposed to map onto a more comprehensive, transdiagnostic models of anhedonia (Husain & Roiser, 2018); for example, reward liking is related to the consummatory phase with positive or negative impact, reward wanting is related to selection between options and initiating and sustaining approach behaviour (incentive motivation) as well as the anticipation and preparation phase, and reward learning is related to learning from outcomes to optimize future decisions. Additionally, it should be noted that overlap does exist across these subtypes; indeed, reward wanting involves valuation and decision-making processes, and all three subtypes involve some representation of the hedonic value of the reward.

## Poor outcomes of anhedonia

Research has increased our understanding of the partially dissociable behavioural deficits underlying anhedonia in MDD. However, common antidepressants, such as selective serotonin reuptake inhibitors (SSRIs) do not ameliorate these behavioural deficits (Argyropoulos & Nutt, 2013; Price, Cole, & Goodwin, 2009), and, conversely, SSRIs have been shown to *blunt* neural responses to rewarding stimuli in healthy controls (McCabe, Mishor, Cowen, & Harmer, 2010). Therefore, those experiencing anhedonia may show a worse response to treatment. Indeed, anhedonia predicts a longer time to remission in adolescents treated with medication switch or medication switch with added cognitive behavioural therapy (CBT; McMakin et al., 2012), and poor antidepressant treatment response in adults (Uher et al., 2012); additionally, objectively measured impairments in reward learning are associated with poorer response to inpatient treatment (Vrieze et al., 2013). Furthermore, anhedonia is associated with both increased severity of depressive symptoms (Gong et al., 2017; Pelizza & Ferrari, 2009) and illness persistence (Spijker, Bijl, de Graaf, & Nolen, 2001). Therefore, to diminish the association between anhedonia, poorer treatment response, and worse illness outcomes, new targeted treatments are required to specifically address anhedonia. Indeed, in pharmacological treatment development, it is helpful to have objective neurobiological markers of successful treatment of the symptom (Krystal et al., 2018). Neuroimaging is a key tool for improving our understanding of the neurobiology of anhedonia in MDD, which is vital for developing these new targeted treatments and neurobiological treatment markers.

## Importance of neuroimaging in anhedonia

Functional magnetic resonance imaging (fMRI) has been used extensively to investigate the neural abnormalities associated with anhedonia in MDD within the frontostriatal reward processing network, which comprises frontal areas such as the ventromedial prefrontal cortex (vmPFC), and orbitofrontal cortex (OFC), and midbrain limbic areas, including the ventral striatum (VS), insula, and thalamus (Haber & Knutson, 2010; Sescousse, Caldu, Segura, & Dreher, 2013). By using fMRI to compare activation and connectivity in the frontostriatal network between MDD patients and controls during reward processing tasks, researchers can assess the neural abnormalities underpinning the behavioural deficits across reward liking, reward wanting, and reward learning, which make up anhedonia in MDD. This empirical data are essential for the development of neurobiological models of anhedonia (Treadway & Zald, 2011).

## Aim of the current study

Functional MRI studies demonstrate that MDD patients show abnormalities in frontostriatal functioning and connectivity during reward processing, associated with the behavioural reward processing deficits making up anhedonia (Admon & Pizzagalli, 2015). Indeed, striatal hypoactivation in response to rewards in MDD has been highlighted by meta-analyses (Keren et al., 2018; Zhang, Chang, Guo, Zhang, & Wang, 2013) and transdiagnostic reviews of the neural basis of reward liking, wanting, and learning deficits (Baskin-Sommers & Foti, 2015; Whitton, Treadway, & Pizzagalli, 2015). Striatal hypoactivation has also been reported for anticipation and receipt of rewards in a review focusing on adolescent MDD (O’Callaghan & Stringaris, 2019). However, complexity and inconsistency in the literature regarding the neurobiological changes associated with each of the three subtypes of anhedonia remains significant. This paper presents an updated and reframed review of neuroimaging studies in MDD across the three subtypes of anhedonia, with reference to more recent comprehensive anhedonia models (Husain & Roiser, 2018). The aim is to further clarify patterns in the data, detail inconsistencies in the literature, understand the limitations of the current evidence base, and make recommendations for future research. The results of this study will be important for improving our understanding of the common and dissociable neural underpinnings of the three subtypes of anhedonia in MDD and elucidating findings from the literature, which will aid in the development of neurobiological models of anhedonia. These, in turn, we hope will be important for developing targeted treatments and revealing neurobiological markers of successful treatment of anhedonia.

## Methodology

English language studies dated from 1992 to August 2019 were included in this systematic review, as the first study using fMRI neuroimaging in humans was published in 1992. Studies were included if they used fMRI to compare participants with current or remitted major depressive disorder (MDD) with controls using reward processing tasks to probe reward liking, reward wanting, and/or reward learning.

**Fig. 1:**
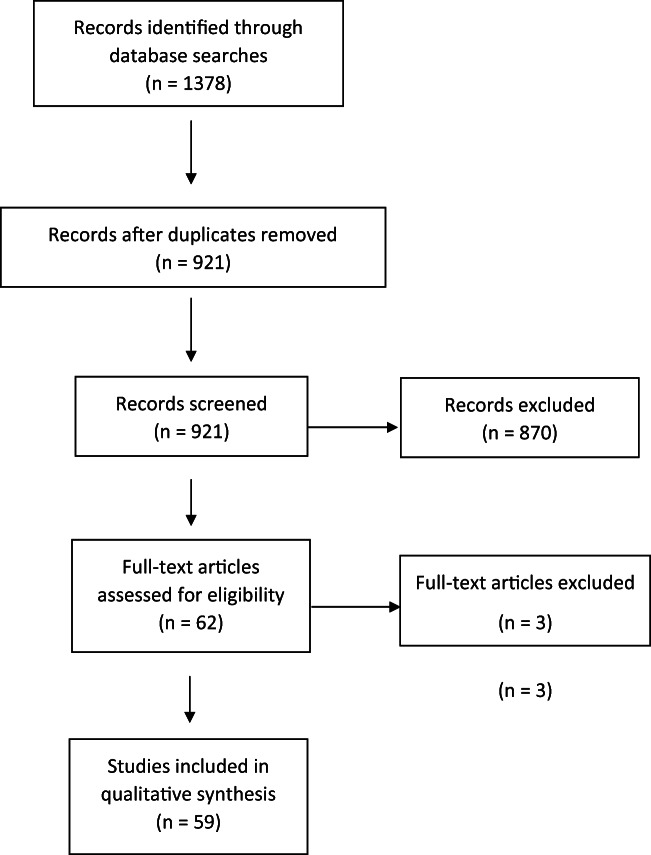
PRISMA flow diagram showing flow of information through the systematic review process, including records identified, screened, included, and excluded

Studies were identified using the keywords ‘reward processing’ or ‘reward’ or ‘anhedonia’; ‘depression’ or ‘major depression’ or ‘major depressive disorder’ or ‘MDD’; ‘neuroimaging’ or ‘fmri’ or ‘fMRI’. The following databases were searched to identify relevant studies: PubMed, The Cochrane Library, PsycINFO, Web of Science. Studies from the database searches were initially screened based on the title and abstract, and then the full text was reviewed for relevant studies. Studies identified by the searches were excluded for the following reasons: using healthy participants rather than participants with current or remitted MDD versus healthy controls, using resting state fMRI rather than fMRI with a reward processing task, observing behaviour rather than using fMRI, and/or using a different imaging technique such as EEG to measure event-related potentials. A PRISMA flow diagram is presented in Fig. 1. Extracted information included the author and date, the neuroimaging technique, the subtype of reward processing studied, the reward processing task used, the sample characteristics, the diagnostic criteria, and the neuroimaging abnormality observed in MDD.

**Fig. 2:**
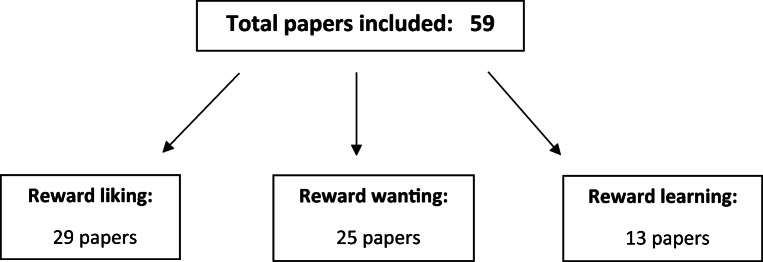
Summary of the total number of studies included and the number of studies identified for each subtype of anhedonia. Six papers were included in both reward liking and reward wanting, and one paper was included in all three subtypes

A total of 59 studies using the following reward processing tasks were included: the monetary incentive delay task (MID), presentation of positive and negative stimuli (including pictorial, word, and oral stimuli, and pleasant music), reward guessing tasks involving choosing between stimuli and receiving a random reward or loss outcome, the Wheel of Fortune (WoF) task, the Effort Expenditure for Reward Task (EEfRT), an effort-based cost-benefit valuation task, Pavlovian, instrumental and reversal learning tasks, probabilistic reward tasks involving choosing between lotteries with varying values and probabilities, and the slot machine task involving receiving unexpected rewards based on the outcome of slot machine spins. Two of the 59 studies identified were meta-analyses. It was recorded whether the study interpreted neuroimaging results from their tasks as relating to reward liking, reward wanting, and/or reward learning processes, and studies were categorized into one or more of the three subtypes based on this. A summary of identified studies for each reward processing subtype is presented in Fig. 2.

## Results

### Reward liking

A total of 29 studies investigating neuroimaging abnormalities in MDD during the experience of reward or loss were identified. These studies used a variety of tasks, including the monetary incentive delay task (MID), presentation of positive and negative stimuli, reward guessing tasks, and the reward delivery phases of an instrumental loss-avoidance win-gain task, a probabilistic reward task, and the wheel of fortune task (WoF). Two meta-analyses were also identified. A summary of identified papers for reward liking is presented in Table 1.

**Table 1:** Papers investigating neuroimaging in major depressive disorder (MDD) within the subtype of reward liking, in the order they appear in the results section

REWARD LIKING					
Author	Reward task	Sample characteristics	Mean depression and anhedonia scores	Diagnostic criteria	Neuroimaging abnormality
Knutson et al., 2008	Monetary incentive delay task (MID)	14 Major Depressive Disorder (MDD) v 12 healthy controls (HCs)	Beck Depression Inventory–II (BDI-II) 25.38 No anhedonia score provided	Met DSM-IV criteria	Recruit ACC more during anticipation of increasing gains, opposite to controls
Pizzagalli et al., 2009	MID	30 unmedicated MDD v 31 HCs	Hamilton Depression Rating Scale (HAM-D) 17.97 BDI-II 27.48 Anhedonia present and assessed using BDI-II subscale, but score not reported	Met DSM-IV criteria	NAc and caudate hypoactivation for rewards, and caudate hypoactivation for losses
Sankar et al., 2019	MID	20 female MDD v 20 HCs	HAM-D 14.88 No anhedonia score provided	Met DSM-IV criteria	Did not show activation in right anterior insula in response to gains and losses, unlike controls
Forbes et al., 2009	Monetary reward guessing task	15 adolescents with MDD v 28 adolescent HCs	No HAM-D or BDI score provided No anhedonia score provided	Diagnosis assessed by K-SADS-PL, confirmed by interview with child psychiatrist	Caudate hypoactivation, and dorsolateral prefrontal cortex (dlPFC) and mPFC hyperactivation for rewards, correlating with lower positive affect
Redlich et al., 2015	Card guessing	33 MDD v 33 bipolar disorder v 34 HCs	HAM-D 24.56 BDI 27.88 Snaith-Hamilton Pleasure Scale (SHAPS) 6.26 (HCs 0.52), binary scoring system where higher scores indicated higher anhedonia	Met DSM-IV criteria	NAc hypoactivation for rewards, and increased coupling with ventral tegmental area (VTA)
Foti et al., 2014	Monetary reward guessing task	24 MDD v 18 HCs	MASQ depression subscale 38.09 MASQ anhedonia subscale 64.36 (HCs 40.00)	Met DSM-IV criteria	VS hypoactivation for reward, correlating with impaired mood reactivity
Satterthwaite et al., 2015	Card guessing	25 MDD v 27 bipolar v 37 HCs	BDI-II 21.75 No anhedonia score provided	Met DSM-IV criteria	Hypoactivation in VS, cingulate and insula for rewards, correlating with depression severity
Steele et al., 2007	Card guessing	15 MDD v 14 HCs	HAM-D 27.5 BDI 36.9 SH 33.8 (HCs 51.9), where higher scores indicated lower anhedonia	Met DSM-IV criteria	ACC hypoactivation for negative feedback, VS hypoactivation for positive feedback
Carl et al., 2016	MID	33 MDD v 20 HCs	BDI-II 25.27 Anhedonia assessed using BDI-II subscale score 4.91	Met DSM-IV criteria	Faster NAc attenuation to rewards
Moses-Kolko et al., 2011	Card guessing	12 post-partum MDD v 12 HCs	HAM-D 21.3 FCPS 126.3 (HCs 139.9)	Met DSM-IV criteria, HAM-D score ≤15 in past month	Faster VS attenuation to rewards
Epstein et al., 2006	Positive and negative word stimuli	10 MDD v 12 HCs	No HAM-D or BDI score provided Anhedonia assessed with 1 question on HAM-D, score not provided	Met DSM-IV criteria	Ventral striatum (VS) hypoactivation for positive stimuli, correlating with anhedonia
Connolly et al., 2015	Affective pictorial stimuli	51 female unmedicated MDD v 61 HCs	IDS-C score 25.43 Anhedonia score from average of two IDS-C items 1.71 (HCs 0.02)	Met DSM-IV criteria	Striatal hypoactivation for affective stimuli, across caudate, putamen and nucleus accumbens (NAc)
Antonesei et al., 2018	Gustatory reward stimuli	26 MDD v 33 HCs	No HAM-D, BDI or anhedonia score provided.	Not reported	Left caudate hypoactivation in response to targets predicting rewarding stimuli
Keedwell et al., 2005	Happy and sad emotional stimuli	12 MDD v 12 HCs	BDI 33.5 Fawcett-Clark Pleasure Scale (FCPS) 63.3 (HCs not provided)	Met ICD-10 criteria	Ventromedial prefrontal cortex (vmPFC) hyperactivation and VS hypoactivation for happy stimuli, correlating with anhedonia
Osuch et al., 2009	Listening to favourite music	16 MDD v 15 HCs	BDI 25.3 SHAPS 36.4 (significantly lower than HCs), where higher SHAPS scores indicated lower anhedonia	Met DSM-IV criteria	Hypoactivation of orbitofrontal cortex (OFC) and VS for music
Jenkins et al., 2018	Listening to preferred music	12 MDD v 10 HCs	HAM-D 15.08 SHAPS 6.67 (HCs 0.00), binary scoring system where higher SHAPS scores indicated higher anhedonia	Met DSM-IV criteria	Faster attenuation of NAc activation
Johnston et al., 2015	Instrumental loss-avoidance and win-gain task	20 treatment resistant MDD v 20 HCs	HAM-D 16.00 BDI-II 32.42 No anhedonia score provided	Clinical diagnosis in tertiary service for treatment resistant MDD	Striatal hyperactivation for rewards, less hippocampal deactivation for losses
Forbes et al., 2006	Probabilistic reward task	14 MDD v 17 HCs, all aged 9–17	No HAM-D or BDI score provided No anhedonia score provided	Diagnosis assessed using K-SADS-PL, met DSM-IV criteria	Hypoactivation of ACC, caudate and OFC, and hyperactivation of amygdala
Keren et al., 2018	Meta-analysis	38 fMRI studies	NA	NA	Striatal hypoactivation for rewards
Zhang et al., 2013	Meta-analysis	22 fMRI studies	NA	NA	Caudate hypoactivation for rewards
McCabe et al., 2009	Sight and flavour of pleasant and aversive foods	13 remitted MDD v 14 HCs	HAM-D 2.3 BDI 5.5 FCPS 118 (HCs 118), no significant difference SHAPS 23 (HCs 19.25), no significant difference	Met DSM-IV criteria for at least 1 past major depressive episode (MDE), no current Axis I psychopathology	VS hypoactivation for pleasant stimuli, and caudate hypoactivation for unpleasant stimuli
Ubl, Kuehner, Kirsch, Ruttorf, Diener, et al., 2015b	Probabilistic reward task	30 unmedicated MDD v 29 HCs	HAM-D 18.40 BDI-II 25.50 SHAPS 42.93 (HCs 49.29), where higher SHAPS scores indicated lower anhedonia	Met DSM-IV criteria	No difference in striatal activation for rewards
Engelmann et al., 2017	Probabilistic reward task	19 unmedicated MDD v 23 HCs	No HAM-D or BDI score provided No anhedonia score provided	Met DSM-IV criteria	Increased coding of losses in anterior insula
Mitterschiffthaler et al., 2003	Positive and negative valenced images	7 females with MDD and high anhedonia v 7 HCs	BDI 33.6 FCPS 2.90 (HCs 4.14)	Met DSM-IV criteria, full criteria met over period of ≥2 years.	Hypoactivation of mPFC, and hyperactivation of inferior frontal cortex, anterior cingulate cortex (ACC), thalamus, putamen and insula for positive images
Kumar et al., 2015	MID	12 MDD v 10 HCs	BDI-II 25.25 SHAPS 5.42 (HCs 0.40), binary scoring system where higher scores indicated higher anhedonia	Met DSM-IV criteria	Hyperactivation of medial prefrontal cortex (mPFC) for rewards under stress, greatest with previous adverse life events
Dichter et al., 2012	MID	19 remitted MDD v 19 HCs	BDI 2.63 No anhedonia score provided	Met DSM-IV criteria for remitted MDD no current Axis I psychopathology	Hypoactivation in OFC, frontal pole, thalamus and insula for rewards
McCabe, 2016	Subjective ratings of oral stimuli	13 remitted MDD v 14 HCs	HAM-D 2.3 BDI 5.5 FCPS 118 (HCs 118), no significant difference SHAPS 23 (HCs 19.25), no significant difference	Met DSM-IV criteria for at least 1 past Major Depressive Episode (MDE), recovery assessed through clinical interview and HAM-D score <8	Negative correlation of dorsomedial prefrontal cortex (dmPFC) with liking of stimuli
Schiller et al., 2013	MID	19 remitted MDD v 19 HCs	BDI-II 2.6 No anhedonia score provided	Met DSM-IV criteria for remitted MDD, no current Axis I psychopathology	Superior frontal and inferior frontal hypoactivation for losses
Morgan et al., 2016	Card guessing	43 boys with history of MDD v 68 with history of other psychiatric illnesses v 55 HCs	MAFQ 6.27 No anhedonia score provided	Diagnosis assessed using K-SADS at ages 8, 10, 11, 12, and using DSM-IV at age 20	Increased connectivity from the mPFC to striatal areas for rewards
Young et al., 2016	Listening to pleasant music	25 MDD v 25 HCs	HAM-D 26.57 Anhedonia subscale of Mood and Anxiety Symptom Questionnaire (MASQ), MASQ-AD 61.81 (HCs 39.27)	Met DSM-IV criteria	Reduced connectivity from posterior vmPFC to other frontostriatal areas, including the OFC, insula, NAc, and VTA, during music, correlating with anhedonia

Using the MID, during which participants are presented with a cue indicating potential gains and losses and then perform a speeded button press to win the gain or avoid the loss (Knutson et al., 2000), studies have observed hypoactivation of the caudate and nucleus accumbens (NAc) hypoactivation in response to rewards in unmedicated MDD participants (Pizzagalli et al., 2009), and hypoactivation of the right anterior insula in response to gains and losses in females with MDD (Sankar et al., 2019).

Using reward guessing tasks, which involve participants choosing between stimuli and receiving a random reward or loss outcome (Delgado, Nystrom, Fissell, Noll, & Fiez, 2000), other studies have found similar patterns in MDD patients during response to rewards, including hypoactivation in the caudate (Forbes et al., 2009), NAc (Redlich et al., 2015), ventral striatum (VS; Foti, Carlson, Sauder, & Proudfit, 2014; Satterthwaite et al., 2015; Steele, Kumar, & Ebmeier, 2007), and insula (Satterthwaite et al., 2015). In one study, caudate hypoactivation correlated with lower positive affect (Forbes et al., 2009), and, in another, VS hypoactivation correlated with impaired mood reactivity (Foti et al., 2014). Moreover, faster attenuation of NAc activity has been reported during response to rewards in MDD using the MID (Carl et al., 2016), and a study in females with postpartum depression using a reward guessing task found faster attenuation of VS activation in response to rewards (Moses-Kolko et al., 2011).

Similar patterns have been found using passive responses to positive and negative stimuli. Studies have observed VS hypoactivation in response to positive word stimuli in MDD (Epstein et al., 2006); hypoactivation across the caudate, putamen, and NAc in response to affective pictures in unmedicated females with MDD (Connolly, Gollan, Cobia, & Wang, 2015); hypoactivation of the left caudate in response to targets predicting rewarding gustatory stimuli (Antonesei, Murayama, & McCabe, 2018); and hypoactivation of the VS in response to happy stimuli (Keedwell, Andrew, Williams, Brammer, & Phillips, 2005) and to favourite music (Osuch et al., 2009). Furthermore, similar to results using both the MID and guessing tasks, it has also been observed that MDD participants have faster attenuation of NAc activation when listening to preferred music (Jenkins et al., 2018). In one study, the reported striatal hypoactivation did not correlate with MDD or anhedonia severity (Connolly et al., 2015), but in other studies, hypoactivation of the VS did correlate with anhedonia levels (Epstein et al., 2006; Keedwell et al., 2005).

Other tasks have also been used to investigate reward liking in MDD, albeit more rarely. Using an instrumental loss-avoidance win-gain task, striatal hypoactivation during response to rewards in participants with treatment-resistant MDD (Johnston et al., 2015). Furthermore, using a probabilistic reward task involving choosing between options with differing reward values and probabilities (Rogers et al., 2003), hypoactivation of the caudate was found in children with MDD in response to rewards (Forbes et al., 2006).

In terms of meta-analyses, one reported striatal hypoactivation in MDD during reward feedback (Keren et al., 2018), and another found specifically caudate hypoactivation during response to rewards (Zhang et al., 2013), both across studies using a variety of reward processing tasks, such as the MID, card guessing, and presentation of positive and negative stimuli (Keren et al., 2018; Zhang et al., 2013). Furthermore, the striatal hypoactivation patterns in response to rewards reported in the above studies have been also been observed in remission, as one study reported those with remitted MDD showed VS hypoactivation for pleasant food stimuli and caudate hypoactivation for unpleasant food stimuli (McCabe, Cowen, & Harmer, 2009).

However, three identified studies observed different patterns of striatal activation in MDD in response to rewards: A study using a reinforcement learning task with unmedicated MDD participants found no striatal hypoactivation in response to rewards (Ubl, Kuehner, Kirsch, Ruttorf, Diener, et al., 2015b); another using a probabilistic reward task showed increased coding of losses in the anterior insula in unmedicated MDD (Engelmann, Berns, & Dunlop, 2017); and an early study actually found hyperactivation of lower limbic areas (including the thalamus, putamen, and insula) in response to positively valanced images (Mitterschiffthaler et al., 2003).

In terms of frontal areas, hyperactivation of the medial prefrontal cortex (mPFC) has been reported in MDD participants during response to rewards on the MID task under stress, and this effect was greatest when individuals had experienced previous adverse life events (Kumar et al., 2015). Similarly, using reward guessing tasks, studies have found vmPFC and dorsolateral prefrontal cortex (dlPFC) hyperactivation in response to rewards in adolescents with MDD, correlating with lower positive affect (Forbes et al., 2009). This time using response to happy stimuli, another study also observed frontal hyperactivation in the ventromedial prefrontal cortex (vmPFC), alongside lower limbic hypoactivation in the VS, in MDD (Keedwell et al., 2005), correlating with anhedonia levels (Keedwell et al., 2005).

In contrast, hypoactivation of the orbitofrontal cortex (OFC) has been observed in MDD participants when listening to their favourite music (Osuch et al., 2009) and in children with MDD during a probabilistic reward task (Forbes et al., 2006). In the latter study, they also observed hypoactivation of the anterior cingulate cortex (ACC) in response to rewards (Forbes et al., 2006). Moreover, cingulate cortex hypoactivation in response to rewards has been reported in MDD participants using reward guessing tasks (Satterthwaite et al., 2015), as well as lower anterior cingulate cortex (ACC) recruitment for unexpected losses (Steele et al., 2007).

During remission of MDD, frontal areas have been found to show a general pattern of hypoactivation (in the OFC and right frontal pole) during response to rewards on the MID (Dichter, Kozink, McClernon, & Smoski, 2012), and a negative correlation between dmPFC activity and liking of an oral stimulus, opposite to healthy controls (McCabe, 2016). Additionally, a study observed hypoactivation of the superior and inferior frontal gyri in response to losses in remitted MDD (Schiller, Minkel, Smoski, & Dichter, 2013).

Two other identified studies in the reward liking subtype assessed frontostriatal connectivity during response to rewards: one study reported that, in a group of male adolescents, those with a history of MDD showed increased frontostriatal connectivity during delivery of rewards on the reward guessing task (Morgan et al., 2016), whereas another found reduced connectivity between the posterior vmPFC and other frontostriatal regions in MDD while listening to pleasant music (Young et al., 2016), which correlated with higher anhedonia levels (Young et al., 2016).

### Reward wanting

A total of 25 papers investigating neuroimaging abnormalities in MDD patients were identified for the reward wanting subtype. In line with recent models of anhedonia, these reward wanting studies were divided into those focusing on the anticipatory phase (reward anticipation) and those looking at selection between reward options and motor mechanisms initiating approach behaviour (incentive motivation; Husain & Roiser, 2018). These studies used a variety of tasks, including the EEfRT an effort-based cost-benefit valuation task; the WoF for incentive motivation; and the MID, reward guessing tasks, and a slot machine task for reward anticipation. One meta-analysis was also identified. A summary of identified papers for reward wanting is presented in Table 2.

**Table 2:** Papers investigating neuroimaging in major depressive disorder (MDD) within the subtype of reward wanting, grouped by incentive motivation and reward anticipation, and in the order they appear in the results section

REWARD WANTING					
*Incentive motivation*					
Author	Reward task	Sample characteristics	Mean depression score	Diagnostic criteria	Neuroimaging abnormality
Yang et al., 2016	Effort expenditure for rewards task (EEfRT)	25 MDD v 25 HCs	HAM-D 27.58 BDI 33.04 SHAPS 34.36 (HCs 21.56), where higher scores indicated higher anhedonia TEPS 63.52 (HCs 91.00)	Met DSM-IV criteria	Caudate hypoactivation during reward selection
Smoski et al., 2009	Wheel of Fortune (WoF)	16 MDD v 15 HCs	HAM-D 23.5 No anhedonia score provided	Met DSM-IV criteria	OFC hyperactivation and dorsal anterior cingulate cortex (ACC) hypoactivation during reward selection; Caudate hypoactivation, but no change in medial prefrontal cortex (mPFC) during reward anticipation
Shad et al., 2011	WoF	22 adolescents with MDD v 22 adolescent HCs	No HAM-D or BDI score provided No anhedonia score provided	Diagnosis assessed using K-SADS-PL	OFC hypoactivation, and right ACC hyperactivation during reward selection
Forbes et al., 2006	Probabilistic reward task	14 MDD v 17 HCs, all aged 9–17	No HAM-D or BDI score provided No anhedonia score provided	Diagnosis assessed using K-SADS-PL, met DSM-IV criteria	OFC hyperactivation during reward selection
Park et al., 2017	Effort-based cost-benefit valuation task	22 MDD v 23 schizophrenia v 31 HCs	HAM-D 15.5 BDI 25.9 Apathy Evaluation Scale (AES) 43.4 (HCs 35.5)	Met DSM-IV criteria	Reduced medial orbitofrontal cortex (OFC)-striatal functional connectivity
*Reward anticipation*					
Author	Reward task	Sample characteristics	Mean depression score	Diagnostic criteria	Neuroimaging abnormality
Arrondo et al., 2015	MID	24 MDD v 22 schizophrenia v 21 HCs	BDI median 32 SHAPS 36 (HCs 24), where higher score indicated higher anhedonia Temporal Experience of Pleasure Scale (TEPS) 53.5 (HCs 80)	Met DSM-IV criteria, confirmed diagnosis using PANSS and Mini-International Psychiatric Inventory	VS hypoactivation during reward anticipation, not correlating with anhedonia
Hagele et al., 2015	MID	24 MDD v 106 other psychiatric illness v 54 HCs	BDI 24.3 No anhedonia score provided	Met ICD-10 and DSM-IV criteria	VS hypoactivation during reward anticipation
Takamura et al., 2017	MID	12 MDD v 12 HCs	HAM-D 20.1 BDI-II 30.8 No anhedonia score provided	Met DSM-IV criteria	Decreased VS and putamen sensitivity to increasing rewards
Stringaris et al., 2015	MID	At baseline, 22 MDD v 101 subthreshold MDD v 123 HCs.	Strengths and Difficulties Questionnaire (SDQ) MDD 16.4 Subclinical MDD 13.9 Coded as having anhedonia is rated by self-report in screening questions of Development and Well-being Assessment (DAWBA)	Met DSM-IV criteria.	VS hypoactivation during reward anticipation in those with current or future subthreshold and clinical MDD, associated with anhedonia scores
Ubl, Kuehner, Kirsch, Ruttorf, Diener, et al., 2015b	Probabilistic reward task	30 unmedicated MDD v 29 HCs	HAM-D 18.40 BDI-II 25.50 SHAPS 42.93 (HCs 49.29), where higher scores indicated lower anhedonia	Met DSM-IV criteria	VS, ACC and OFC hypoactivation during reward anticipation
Hamilton et al., 2018	MID	16 MDD v 14 HCs	HAM-D 13.6 BDI-II 26.27 SHAPS 49 (HCs 64), where higher scores indicated lower anhedonia	Met DSM-5 criteria	Lower dopamine activity in the VS and right dorsal striatum, associated with lower connectivity to cortical targets
Misaki et al., 2016	MID	44 MDD v 45 HCs	HAM-D 17.3 SHAPS 28.9 (HCs 18.3), where higher scores indicated higher anhedonia	Met DSM-IV criteria	Nucleus accumbens (NAc) hypoactivation during reward anticipation, correlating with anhedonia
Pizzagalli et al., 2009	MID	30 unmedicated MDD v 31 HCs	HAM-D 17.97 BDI-II 27.48 Anhedonia present and assessed using BDI-II subscale, but score not reported	Met DSM-IV criteria	Putamen hypoactivation during reward anticipation
Olino et al., 2011	Card guessing	10 MDD v 16 HCs, aged 8–16 years	No HAM-D or BDI score provided No anhedonia score provided	Diagnosis assessed using K-SADS	Ventral striatum (VS) hypoactivation during reward anticipation
Insel et al., 2018	Card guessing	56 MDD v 56 HCs, females aged 15–20 years	No HAM-D or BDI score provided No anhedonia score provided	Met DSM-IV criteria	No increase in striatal recruitment for higher magnitude rewards
Forbes et al., 2009	Monetary reward guessing task	15 adolescents with MDD v 28 adolescent HCs	No HAM-D or BDI score provided No anhedonia score provided	Diagnosis assessed by K-SADS-PL, confirmed by interview with child psychiatrist	Striatal hypoactivation, and dorsolateral prefrontal cortex (dlPFC) and mPFC hyperactivation during reward anticipation
Zhang et al., 2013	Meta-analysis	22 fMRI studies	NA	NA	Caudate hypoactivation, and ACC and middle frontal gyrus hyperactivation during reward anticipation
Gorka et al., 2014	Slot machine task	9 MDD v 13 MDD with co-morbid panic disorder v 18 HCs	HAM-D 26.3 for MDD HAM-D 28.2 for MDD with co-morbid panic disorder No anhedonia score provided	Met DSM-IV criteria	Hyperactivation of dorsal ACC during reward anticipation
Smoski et al., 2011	MID	9 MDD v 13 HCs	BDI-II 16.7 No anhedonia score provided	Met DSM-IV criteria	OFC, subcallosal cingulate and paracingulate hypoactivation during reward anticipation
Chase et al., 2013	Card guessing	40 MDD v 23 bipolar disorder v 37 HCs	HAM-D 26.63 No anhedonia score provided	Met DSM-IV criteria	Hyperactivation of ACC during reward anticipation
Dichter et al., 2012	MID	19 remitted MDD v 19 HCs	BDI 2.63 No anhedonia score provided	Met DSM-IV criteria for remitted MDD, no current Axis I psychopathology	Hyperactivation of ACC, right midfrontal gyrus and cerebellum during reward anticipation
Ubl, Kuehner, Kirsch, Ruttorf, Flor, et al., 2015a	MID	23 remitted MDD v 23 HCs	HAM-D 3.23 BDI-II 2.04 SHAPS 49.10 (HCs 49.87), no significant difference	Met DSM-IV criteria for 2 or more past MDEs, no current MDE or dysthymia	Hyperactivation in frontostriatal regions during reward anticipation
Schiller et al., 2013	MID	19 remitted MDD v 19 HCs	BDI-II 2.6 No anhedonia score provided	Met DSM-IV criteria for remitted MDD, no current Axis I psychopathology	Superior frontal gyrus hypoactivation during loss anticipation
Manelis et al., 2016	Card guessing	46 MDD v 36 bipolar v 42 HCs	HAM-D 26.97 No anhedonia score provided	Met DSM-IV criteria	Increased frontostriatal connectivity during loss anticipation, and decreased connectivity during reward anticipation

#### Incentive motivation

Using the EEfRT (Treadway et al., 2009), which assesses neural responses during selection between reward options with varying reward values, probabilities, and physical effort requirements, one study found caudate and superior temporal gyrus hypoactivation in MDD participants for high reward and high probability choices, respectively (Yang et al., 2016).

In terms of more frontal areas, one study used the WoF task, during which participants select between two reward options with varying reward values and probability (Ernst et al., 2004) to assess neural responses during reward selection. Indeed, this study found OFC hyperactivation in MDD (Smoski et al., 2009), as did a study using a probabilistic reward task (Forbes et al., 2006). However, contrastingly, a later study found the opposite in adolescents with MDD, observing OFC hypoactivation during reward selection on this same task (Shad et al., 2011). Furthermore, this study also reported ACC hyperactivation during reward selection (Shad et al., 2011).

One identified study investigated connectivity during reward selection in MDD using an effort-based cost-benefit valuation task, involving pressing a button to turn off light bulbs a varying number of times to attain a reward (Park, Lee, Kim, Kim, & Koo, 2017); this study observed reduced functional connectivity between the medial OFC and the striatum during this task (Park et al., 2017).

#### Reward anticipation

Using the MID to assess neural responses during anticipation of rewards following cue presentation, three fMRI studies observed VS hypoactivation during reward anticipation in MDD (Arrondo et al., 2015; Hagele et al., 2015; Takamura et al., 2017). Additionally, VS hypoactivation during reward anticipation has been shown to be associated with both current and future subthreshold and clinical MDD (Stringaris et al., 2015) and was observed in a sample of unmedicated MDD participants (Ubl, Kuehner, Kirsch, Ruttorf, Diener, et al., 2015b). One identified study with MDD participants utilized simultaneous fMRI and positron emission tomography (PET) alongside the MID and observed lower VS and right dorsal striatum dopamine activity alongside lower connectivity with cortical targets (Hamilton et al., 2018). The MID involves components of various reward processes, but striatal dopamine activity is associated with reward coding during anticipation (Abler, Walter, Erk, Kammerer, Spitzer, 2006).

Other studies using the MID in MDD participants have also found patterns of striatal hypoactivation during reward anticipation, in both the NAc (Misaki, Suzuki, Savitz, Drevets, & Bodurka, 2016) and the putamen (Pizzagalli et al., 2009; Takamura et al., 2017), with one study using an unmedicated MDD sample (Pizzagalli et al., 2009). In agreement with studies using the MID, studies using the card guessing task have reported VS hypoactivation during reward anticipation in children with MDD (Olino et al., 2011) and lower striatal reactivity to rewards in adolescents (Insel, Glenn, Nock, & Somerville, 2018). Correlations between striatal hypoactivation during reward anticipation and anhedonia scores have been noted by two identified studies, in both the VS (Stringaris et al., 2015) and the NAc (Misaki et al., 2016). However, one study found no association between VS hypoactivation and anhedonia levels (Arrondo et al., 2015).

Other identified studies reported findings across frontostriatal areas. Firstly, striatal hypoactivation has been observed alongside dlPFC and medial prefrontal cortex (mPFC) hyperactivation during reward anticipation in adolescents with MDD on a monetary reward guessing task, correlating with lower positive affect (Forbes et al., 2009). Additionally, a meta-analysis identified a pattern of middle frontal gyrus and anterior cingulate cortex (ACC) hyperactivation, and caudate hypoactivation, during reward anticipation in MDD (Zhang et al., 2013), on tasks including the MID, card guessing, and WoF (Zhang et al., 2013).

In agreement with the ACC hyperactivation noted by the above meta-analysis (Zhang et al., 2013), one identified study, using a passive slot machine task, observed dorsal ACC hyperactivation during reward anticipation in MDD (Gorka et al., 2014), and another, using the MID, found that MDD participants showed increasing ACC activation during anticipation of increasing gains, opposite to controls. However, another study using the MID found paracingulate and subcallosal cingulate hypoactivation during reward anticipation in MDD (Smoski, Rittenberg, & Dichter, 2011), and a study using the card guessing task observed ACC hypoactivation during the reward anticipation phase in MDD (Chase et al., 2013). Further to this, in a study using a sample of unmedicated MDD participants, VS hypoactivation was observed alongside hypoactivation of the OFC and ACC (Ubl, Kuehner, Kirsch, Ruttorf, Diener, et al., 2015b).

In studies using participants with remitted MDD, hyperactivation of the ACC and right midfrontal gyrus has been observed during anticipation of rewards on the card guessing task (Dichter et al., 2012), and hyperactivity across the frontostriatal network has also been observed during reward anticipation on the MID (Ubl, Kuehner, Kirsch, Ruttorf, Flor, et al., 2015a). However, during anticipation of losses rather than gains, another study found superior frontal gyrus hypoactivity in rMDD (Schiller et al., 2013).

The review identified one study investigating frontostriatal connectivity during reward anticipation in MDD using a card guessing paradigm (Manelis et al., 2016), and this study reported lower frontostriatal connectivity during reward anticipation and higher connectivity during loss anticipation in MDD (Manelis et al., 2016).

### Reward learning

A total of 13 papers were identified investigating neuroimaging abnormalities in MDD during both reward learning tasks and receipt of unexpected rewards and losses. These studies used a variety of tasks, including reward learning tasks (Pavlovian, instrumental, reversal learning), the MID, reward guessing tasks, a probabilistic reward task, and the slot machine task. A summary of identified papers for reward learning is presented in Table 3.

**Table 3:** Papers investigating neuroimaging in major depressive disorder (MDD) within the subtype of reward learning, in the order they appear in the results section

REWARD LEARNING					
Author	Reward task	Sample characteristics	Mean depression score	Diagnostic criteria	Neuroimaging abnormality
Kumar et al., 2008	Pavlovian reward learning task	15 MDD v 18 HCs	HAM-D 23.2 BDI 22.9 Snaith Hamilton Hedonia Scale (SH) 35.0 (HCs 51.7), where higher scores indicated lower anhedonia	DSM-IV diagnosis assessed by treating consultant and 1 author, symptom duration >3 months	Reduced prediction error (PE) during learning in ventral striatum (VS), cingulate, midbrain, and hippocampus, correlating with illness ratings
Gradin et al., 2011	Instrumental reward learning task	15 MDD v 14 schizophrenia v 17 HCs	HAM-D 23.2 BDI 22.93 BDI anhedonia score from 4 questions 6.27 (HCs 0.71)	DSM-IV diagnosis assessed by clinical assessment and psychiatric interview by 1 author and consultant psychiatrist	Reduced PE during learning in striatum, caudate, and nucleus accumbens (NAc), correlating with anhedonia
Kumar et al., 2018	Instrumental reward learning task	25 unmedicated MDD v 26 HCs	HAM-D 17.27 BDI 26.6 SHAPS 33.40 (HCs 18.6), where higher score indicated higher anhedonia	Met DSM-IV criteria	Blunted PE in striatum and reduced ventral tegmental area (VTA)-striatal connectivity in response to feedback
Rothkirch et al., 2017	Instrumental reward learning task	28 unmedicated MDD v 30 HCs	HAM-D 22.5 BDI 33.0 SHAPS 5.60 (HCs 0.33), binary scoring system where higher score indicated higher anhedonia	Met DSM-IV criteria	Reduced PE in medial orbitofrontal cortex (OFC), negative correlation between VS PE and anhedonia severity
Geugies et al., 2019	Pavlovian reward learning task	36 remitted MDD v 27 HCs	HAM-D 3 (median) SHAPS 24 (HCs 17) median score, where higher scores indicated higher anhedonia	Met DSM-IV criteria for recurrent depression; stable remission defined as HAM-D score ≤7 for 8 subsequent weeks	Reduced PE in ventral tegmental area (VTA), associated with higher anhedonia levels in remitted MDD.
Ubl, Kuehner, Kirsch, Ruttorf, Diener, et al., 2015b	Probabilistic reward task	30 unmedicated MDD v 29 HCs	HAM-D 18.40 BDI-II 25.50 SHAPS 42.93 (HCs 49.29), where higher scores indicated lower anhedonia	Met DSM-IV criteria	Reduced PE in anterior cingulate cortex (ACC) and amygdala for rewards, increased PE in VS for losses
Greenberg et al., 2015	Card guessing	148 unmedicated MDD v 31 HCs	HAM-D 26.52 SHAPS 33.46 (HCs 20.52), where higher scores indicated higher anhedonia	Met DSM-IV criteria	No inverse relationship between reward expectancy and PE in ventral striatum (VS)
Rutledge et al., 2017	Probabilistic reward task	32 MDD v 20 HCs	HAM-D 16.6 No anhedonia score provided	Patients receiving treatment based on primary MDD diagnosis	No difference in PE signals in VS
Segarra et al., 2016	Slot machine task	24 MDD v 21 schizophrenia, v 21 HCs	BDI 32.62 SHAPS 33.42 (HCs 23.38), where higher scores indicated higher anhedonia	Met DSM-IV criteria	OFC, VS, insula, and thalamus hypoactivation for unexpected rewards (interpreted as positive reward prediction error)
Steele et al., 2004	Card guessing	15 MDD with 15 HCs	HAM-D 27.3 BDI 36.9 No anhedonia score provided	Met DSM-IV criteria	Increased cingulate and parahippocampus PE signalling for unexpected losses, correlating with depression scores
Robinson et al., 2012	Reversal learning	13 unmedicated MDD v 14 HCs	HAM-D 20 No anhedonia score provided	Met DSM-IV criteria	Reduced PE in VS for reward feedback, but not punishment feedback
Hall et al., 2014	Reversal learning	29 MDD v 25 HCs	HAM-D 17.93 first episode of depression HAM-D 9.4 multiple episodes of depression No anhedonia score provided	Met DSM-IV criteria	NAc and ventromedial prefrontal cortex (vmPFC) hypoactivity during reversal learning feedback
Liu et al., 2017	Instrumental reward learning task	24 unmedicated MDD v 21 HCs	HAM-D 24.05 SHAPS 28.5 (HCs 23.6), where higher scores indicated higher anhedonia TEPS 72.6 (HCs 81.9)	Met DSM-IV criteria	Trend towards left habenula hypoactivation in response to losses

The majority of identified studies in the reward learning subtype calculated prediction errors in response to reward feedback, and four of these studies calculated prediction errors during learning tasks, including Pavlovian, instrumental, and reversal learning. Using a Pavlovian learning task, a study found blunted prediction error signalling in the VS in MDD during reward learning, correlating with illness ratings (Kumar et al., 2008). Additionally, using instrumental learning tasks, fMRI studies have found similar blunting of prediction error signalling, this time in the caudate and NAc (Gradin et al., 2011), as well as in the striatum in unmedicated MDD (Kumar et al., 2018). Furthermore, again using an instrumental learning task, another fMRI study observed a negative correlation between VS prediction error signalling and anhedonia severity (Rothkirch, Tonn, Köhler, & Sterzer, 2017). This pattern has also been observed in remission, as a study using a Pavlovian learning task found reduced prediction errors in the ventral tegmental area (VTA), associated with higher anhedonia levels (Geugies et al., 2019).

Similar findings have been observed across other tasks; using the MID to assess neural activity as a function of prediction error signals for unexpected rewards and losses, one study found blunted reward-related prediction error signalling and potentiated loss-related prediction error signalling in the VS in unmedicated MDD participants (Ubl, Kuehner, Kirsch, Ruttorf, Diener, et al., 2015b). Furthermore, a study using a card guessing task observed that those with unmedicated MDD did not show the normal inverse association between reward expectancy and VS prediction error signals (Greenberg et al., 2015). However, a study using a probabilistic reward task, involving choosing between lotteries with varying monetary values and probabilities to assess neural responses during value-based decision-making, observed no difference in VS prediction error signals in MDD participants (Rutledge et al., 2017).

In terms of other brain regions, one study calculating prediction errors using an instrumental learning task found reduced reward prediction errors in the medial OFC during in unmedicated MDD (Rothkirch et al., 2017), alongside reduced VS prediction error signals (Rothkirch et al., 2017). Another study used a slot machine task, where participants are presented with a slot machine with two reels and, when the images on the reels in the centre of view match, the participant wins a financial reward; on 50% of trials, participants can choose the centre image of the left wheel, and in 50% of trials the computer chooses (Segarra et al., 2016). This study observed hyposensitivity of prediction errors for unexpected rewards in the frontostriatal network, including the OFC, VS, insula, and thalamus in MDD (Segarra et al., 2016). Lastly, for unexpected losses rather than gains, an early study using a card guessing paradigm found increased prediction error signalling for unexpected losses in the ACC in MDD, correlating with MDD severity (Steele, Meyer, & Ebmeier, 2004).

Three other identified studies did not calculate prediction errors, but instead investigated neural responses to feedback during learning tasks. An fMRI study using a reversal learning paradigm found VS hypoactivity in response to positive feedback in unmedicated MDD, but did not find abnormal VS activation for negative feedback (Robinson, Cools, Carlisi, Sahakian, & Drevets, 2012). Furthermore, another study using reversal learning observed hypoactivation of frontostriatal regions, including the NAc and vmPFC, during reinforcement contingency changes, correlating with lifetime disease burden (Hall, Milne, & Macqueen, 2014). Finally, one study observed a trend towards left habenula hypoactivation in response to losses during an instrumental learning task in MDD, but this did not reach significance (Liu, Valton, Zhu, & Roiser, 2017).

## Discussion

The aim of this review was to better characterize the neurobiology of anhedonia in MDD; anhedonia was conceptualized as comprising deficits across three partially separable subtypes of reward processing: reward liking, reward wanting, and reward learning (Admon & Pizzagalli, 2015; Rømer Thomsen et al., 2015; Treadway & Zald, 2011), with consideration to how these map onto recent, transdiagnostic models of anhedonia (Husain & Roiser, 2018). The identified studies showed both common and dissociable neural underpinnings for each subtype. For reward liking and reward wanting (including reward anticipation and incentive motivation), studies observed striatal hypoactivation, alongside hypoactivation and hyperactivation across various frontal regions, and, for reward learning, studies observed blunted frontostriatal network sensitivity in response to unexpected rewards and positive feedback, but no abnormality in response to unexpected losses or negative feedback.

### Reward liking

Reward liking represents the consummatory stage of reward processing, involving positive or negative hedonic impact (Berridge & Robinson, 2003; Husain & Roiser, 2018; Rømer Thomsen et al., 2015; Treadway & Zald, 2011). The most consistent neuroimaging abnormality associated with reward liking was striatal hypoactivation, reported to be observed in the VS/NAc (Carl et al., 2016; Connolly et al., 2015; Epstein et al., 2006; Foti et al., 2014; Jenkins et al., 2018; Keedwell et al., 2005; Moses-Kolko et al., 2011; Osuch et al., 2009; Pizzagalli et al., 2009; Redlich et al., 2015; Satterthwaite et al., 2015; Steele et al., 2007), caudate (Antonesei et al., 2018; Connolly et al., 2015; Forbes et al., 2006; Forbes et al., 2009; Pizzagalli et al., 2009; Zhang et al., 2013), putamen (Connolly et al., 2015), and right anterior insula (Sankar et al., 2019).

Six of these studies reported no anhedonia levels in the participants, but 11 identified higher anhedonia levels in the MDD participants using a variety of anhedonia measures, indicating their ability to inform our understanding of anhedonia. Additionally, striatal hypoactivation has been shown to correlate with anhedonia (Epstein et al., 2006; Keedwell et al., 2005), although one study did not find this association (Connolly et al., 2015). These three studies all used different methods of assessing anhedonia; the study finding no correlation used the average of two items of the Inventory of Depressive Symptomology–Clinician-Rated (IDS-C; Connolly et al., 2015), and the studies finding correlations used one item from the Hamilton Anxiety and Depression Scale (HAM-D; Epstein et al., 2006) and the Fawcett-Clark Pleasure Scale (FCPS; Keedwell et al., 2005). Since two studies supported a correlation, and one used the FCPS—which has more items and is validated for measuring anhedonia (Rizvi, Pizzagalli, Sproule, & Kennedy, 2016)—it seems the weight of evidence supports a correlation between striatal hypoactivation and anhedonia severity in MDD.

Indeed, striatal hypoactivation during reward liking is a robust finding, as it has also been observed across heterogeneous tasks (MID, presentation of positive stimuli, card guessing). It must be noted that the MID contrast for specific reward processing phases is difficult to isolate completely, meaning these results may also include some signals related to other phases e.g. prediction error for unexpected rewards. However, similar findings also being observed with the use of passive tasks (presentation of positive stimuli) is important, as these tasks could be argued to have less interference from other reward processing phases (such as reward selection and reward learning).

Striatal hypoactivation associated with reward liking deficits in MDD is consistent with the neurobiology of reward processing, as the striatum plays a key role in hedonic processing (Admon & Pizzagalli, 2015; Berridge and Kringelbach, 2013). Furthermore, striatal hypoactivation during reward liking may be associated with abnormal opioid signalling, as opioid signalling in the striatum mediates our core liking responses (Berridge & Kringelbach, 2008, 2013; Kelley et al., 2002), and is dysfunctional in MDD (Kennedy, Koeppe, Young, & Zubieta, 2006).

However, striatal hypoactivation could be caused by antidepressants, as the SSRI citalopram has been shown to blunt VS activation for pleasant gustatory stimuli in healthy participants (McCabe et al., 2010), and paroxetine blunts striatal activation for erotic stimuli in MDD (Abler, Gron, Hartmann, Metzger, & Walter, 2012). Concurring with these medication effects, one study found no striatal hypoactivation during reward liking when using an unmedicated MDD sample (Ubl, Kuehner, Kirsch, Ruttorf, Diener, et al., 2015b). In contrast, though, two others did observe striatal hypoactivation in unmedicated samples (Connolly et al., 2015; Pizzagalli et al., 2009). Interestingly, the study finding no evidence of striatal hypoactivation (Ubl, Kuehner, Kirsch, Ruttorf, Diener, et al., 2015b) and the one presented by Pizzagalli and colleagues—which did report striatal hypoactivation—both had a sample size of 30 unmedicated individuals with MDD, and the HAM-D average scores were similar (Pizzagalli et al., 2009; Ubl, Kuehner, Kirsch, Ruttorf, Diener, et al., 2015b). Furthermore, this striatal hypoactivation has been shown to persist into unmedicated remission (McCabe et al., 2009), together suggesting medication is unlikely to be the primary cause of striatal hypoactivation during reward liking in MDD.

In the frontal cortex, hyperactivation in the vmPFC (Keedwell et al., 2005), mPFC (Forbes et al., 2009; Kumar et al., 2015), and dlPFC (Forbes et al., 2009) has been observed during reward liking in MDD across three heterogeneous reward processing tasks (MID, card guessing, and presentation of positive and negative stimuli). Although sample sizes were limited to only 12 MDD participants (Keedwell et al., 2005; Kumar et al., 2015) and 15 MDD participants (Forbes et al., 2009), the studies do span an age range from adolescents (Forbes et al., 2009) to adults (Keedwell et al., 2005; Kumar et al., 2015). One of the three studies reported anhedonia scores, indicating significantly higher anhedonia scores in MDD than controls using the SHAPS (Kumar et al., 2015), thus providing preliminary evidence for the relationship between this activation pattern and anhedonia in MDD. Additionally, a pattern of mPFC hyperactivation and striatal hypoactivation in the reward liking subtype of anhedonia is consistent with a recent optogenetics study, which found that stimulating mPFC hyperactivation inhibits striatal responses to rewards, and thus induces an anhedonic phenotype in rats (Ferenczi et al., 2016).

However, distinct frontal regions have dissociable functions in reward processing (Der-Avakian & Markou, 2012), and studies have also found frontal hypoactivation during reward liking, in both the cingulate cortex (Satterthwaite et al., 2015) and OFC (Forbes et al., 2006; Osuch et al., 2009). Again, studies used a variety of reward processing tasks (passive listening to favourite music and active card guessing and probabilistic reward tasks) and an age range including children/adolescents aged 9–17 (Forbes et al., 2006) and adults (Osuch et al., 2009; Satterthwaite et al., 2015). Additionally, one study reported significantly higher anhedonia levels in MDD versus healthy controls using the SHAPS (Osuch et al., 2009), and the probabilistic reward task used by Forbes and colleagues allowed separation of the consummatory reward liking phase from other phases e.g. selection between reward options (Forbes et al., 2006), indicating the usefulness of these results for understanding the reward liking component of anhedonia. This pattern of OFC hypoactivation also continues into remission (Dichter et al., 2012) and is consistent with the role of this area in mediating our conscious experience of rewards (Berridge & Kringelbach, 2008; Kringelbach, 2005; Kringelbach & Berridge, 2010). Therefore, taken together, OFC hypoactivation, alongside striatal hypoactivation and mPFC hyperactivation, is likely to be a neural underpinning of reward liking deficits in MDD.

### Reward wanting

In the anticipatory phase of reward processing, striatal hypoactivation is the most consistent neuroimaging abnormality observed in the VS (Arrondo et al., 2015; Hagele et al., 2015; Insel et al., 2018; Olino et al., 2011; Takamura et al., 2017; Ubl, Kuehner, Kirsch, Ruttorf, Diener, et al., 2015b), NAc (Misaki et al., 2016), caudate (Smoski et al., 2009; Zhang et al., 2013), and putamen (Pizzagalli et al., 2009; Takamura et al., 2017). This appears to be a relatively robust neural basis of reward anticipation deficits in MDD, as it is observed across tasks (WoF, MID, card guessing). Additionally, five out of the 10 studies identified higher anhedonia levels in the MDD participants versus controls (Arrondo et al., 2015; Misaki et al., 2016; Pizzagalli et al., 2009; Ubl, Kuehner, Kirsch, Ruttorf, Diener, et al., 2015b). In addition, this pattern has been shown to correlate with anhedonia (Misaki et al., 2016); however, another study using the same task and also measuring anhedonia using the SHAPS (albeit with a smaller sample size of 24 MDD participants compared with 44 in the former study) did not find this correlation (Arrondo et al., 2015).

There were only two reports of striatal abnormalities during selection between reward options and initiating approach behaviour (incentive motivation), but hypoactivation was observed in the caudate during the EEfRT in MDD in a study identifying significantly higher levels of anhedonia in MDD versus controls with the SHAPS (Yang et al., 2016) and reduced medial OFC to striatal connectivity was observed during an effort-based reward task (Park et al., 2017). Therefore, striatal hypoactivation appears to underpin reward wanting deficits across both reward anticipation and selection.

Striatal hypoactivation in reward wanting deficits may be associated with abnormal dopamine signalling in this region in MDD, as modulating dopamine transmission using amisulpride in healthy individuals is associated with striatal alterations—for example, putamen, NAc (Metzger, Wiegers, Walter, Abler, & Graf, 2015), and dopaminergic activity in the striatum is involved in coding reward expectancy during anticipation (Abler et al., 2006), and driving incentive motivation (Bardgett, Depenbrock, Downs, Points, & Green, 2009; Denk et al., 2005; Salamone, Correa, Farrar, & Mingote, 2007; Salamone, Correa, Nunes, Randall, & Pardo, 2012). Indeed, midbrain dopamine function is dysfunctional in MDD (Dailly, Chenu, Renard, & Bourin, 2004; Dunlop & Nemeroff, 2007; Nestler & Carlezon, 2006), and amisulpride enhancement of dopamine transmission in MDD normalizes striatal hypoactivation during reward processing (Admon et al., 2017). Additionally, the identified study using fMRI and PET observed reduced VS dopamine activity in MDD during the MID (Hamilton et al., 2018). The MID does involve various reward processing components (e.g., reward liking, reward anticipation), and these aspects are closely related in time during this task. However, taken together, the evidence suggests a link between dysfunctional dopamine signalling in MDD and reward wanting deficits.

In the frontal cortex during reward anticipation, mPFC and dlPFC hyperactivation was observed in one study (Forbes et al., 2009), as well as middle frontal gyrus hyperactivation in a meta-analysis (Zhang et al., 2013). Aa further study found no abnormality in mPFC activation (Smoski et al., 2009); however, this study used a sample of 16 participants compared with the 341 MDD participants identified in the meta-analysis (Zhang et al., 2013). Since a limited number of studies present varying abnormalities across frontal regions, it is difficult to form a clear picture of frontal abnormalities during reward anticipation, but there does appear to be a trend towards hyperactivation in certain frontal regions—for example, middle frontal gyrus (Zhang et al., 2013). In terms of the OFC, hypoactivation has been reported in both MDD (Smoski et al., 2011; Ubl, Kuehner, Kirsch, Ruttorf, Diener, et al., 2015b) and remitted MDD (Ubl, Kuehner, Kirsch, Ruttorf, Flor, et al., 2015a). It should be noted that one study used a sample size of only none MDD participants with a relatively low average BDI-II score of 16.7 (Smoski et al., 2011), but the evidence is strengthened by the use of unmedicated participants with significantly higher anhedonia levels than controls (Ubl, Kuehner, Kirsch, Ruttorf, Diener, et al., 2015b) and remitted participants (Ubl, Kuehner, Kirsch, Ruttorf, Flor, et al., 2015a) in the other studies detecting this abnormality. Therefore, there is evidence suggesting OFC hypoactivation is a neural underpinning of reward anticipation deficits in MDD.

In terms of incentive motivation, one study using the WoF observed OFC hyperactivation (Smoski et al., 2009), whereas another using the WoF observed hypoactivation (Shad et al., 2011), and one study using a probabilistic reward task also observed OFC hypoactivation (Forbes et al., 2006). It should be noted that the two studies observing OFC hypoactivation both used child and adolescent populations (Forbes et al., 2006; Shad et al., 2011), whereas the study observing hyperactivation used an adult population (Smoski et al., 2009). This could have had an impact on the inconsistencies observed here, since adolescents and adults show differential neural engagement patterns during reward processing (Eshel, Nelson, Blair, Pine, & Ernst, 2007; Geier et al., 2010; Silverman, Jedd, & Luciana, 2015). Overall, the findings suggest that some form of OFC abnormality (potentially hypoactivation in adolescents and hyperactivation in adults) is associated with reward selection deficits in MDD, although none of these studies provided anhedonia scores, making it difficult to ascertain how these activation patterns relate to anhedonia. However, an OFC abnormality is consistent with the neurobiology of reward processing, as the WoF task consistently activates the OFC (Smith et al., 2009), and the OFC codes the relative values of reward options during decision-making (Der-Avakian & Markou, 2012; Grabenhorst & Rolls, 2011; Hornak et al., 2004). In future, further replication is required to assess the potentially separable OFC abnormalities underpinning adolescent and adult incentive motivation deficits in MDD.

In terms of cingulate cortex abnormalities during reward anticipation, both ACC hypoactivation (Chase et al., 2013), and paracingulate hypoactivation (Smoski et al., 2011) have been observed using the card guessing task and MID, respectively, whereas ACC hyperactivation has been reported by two other studies (Gorka et al., 2014; Knutson, Bhanji, Cooney, Atlas, & Gotlib, 2008). One of these studies also used the MID (Knutson et al., 2008), but the other used a more passive slot machine task (Gorka et al., 2014). The use of this passive rather than active task (slot machine versus card guessing and MID) by Gorka and colleagues could have impacted on the inconsistency in results, as the task did not demand a selection and choice behaviour from the participant prior to anticipating the reward (Gorka et al., 2014), thus potentially better isolating the reward anticipation component. Overall, the weight of evidence appears to support ACC hyperactivation associated with reward anticipation in MDD, especially as it has also been supported by a meta-analysis (Zhang et al., 2013) and has been found to continue into remission (Dichter et al., 2012). However, it is evident that these cingulate abnormalities require further investigation to overcome inconsistency in results.

Inconsistent results have been identified for cingulate abnormalities during incentive motivation, ACC hyperactivation has been identified in MDD (Shad et al., 2011), as has dorsal ACC hypoactivation (Smoski et al., 2009), with both studies using the WoF task (Shad et al., 2011; Smoski et al., 2009). Both also used a similar number of trials—four runs of 46 trials (Smoski et al., 2009) and four runs of 39 trials (Shad et al., 2011)—and similar monetary compensation following the task, but reported different run lengths of 12 minutes (Smoski et al., 2009) and 7.8 minutes (Shad et al., 2011). Inconsistency may have arisen in part through minor methodological differences, but also because the former used an adolescent population (Shad et al., 2011); adults and adolescents have different neural engagement patterns during reward processing, as mentioned above (Geier et al., 2010; Silverman et al., 2015), and, importantly, show different patterns of dorsal ACC recruitment on this task (Eshel et al., 2007). Overall, there are ACC abnormalities associated with incentive motivation deficits in MDD, but these may be specific to adult versus adolescent populations and require further replication to be more comprehensively understood.

### Reward learning

For learning from reward outcomes , there was a pattern of blunted striatal prediction error signalling for positive feedback on learning tasks (Geugies et al., 2019; Gradin et al., 2011; Kumar et al., 2018; Kumar et al., 2008), and lack of inverse relationship between unexpected rewards and prediction error signalling in the VS on a card guessing task (Greenberg et al., 2015). All studies here identified higher anhedonia scores in MDD participants than in healthy controls, using the SHAPS (Geugies et al., 2019; Greenberg et al., 2015; Kumar et al., 2018; Kumar et al., 2008) and a subscale of the BDI (Gradin et al., 2011), thus supporting the relationship between blunted striatal prediction error signalling and anhedonia. Furthermore, blunted VS prediction errors have been shown to correlate with increased anhedonia severity in unmedicated MDD participants (Rothkirch et al., 2017). Although blunted striatal prediction errors were not observed during a probabilistic reward task (Rutledge et al., 2017), patients in this study had a lower average HAM-D score than in those studies finding blunted striatal prediction errors.

Striatal hypoactivation may represent blunted dopamine signalling, because midbrain dopamine neurons projecting to the striatum code prediction errors when reward feedback is better or worse than expected (Abler et al., 2006; Bayer & Glimcher, 2005; Schultz, 1997, 1998), which is essential for reinforcement learning (Glimcher, 2011). Two other studies did not compute prediction error signals, but did report striatal hyposensitivity for unexpected positive feedback (Robinson et al., 2012; Segarra et al., 2016). Therefore, despite heterogeneity in terms of calculating striatal prediction errors, the evidence across both learning tasks and tasks delivering unexpected rewards suggests blunted striatal sensitivity to reward feedback may be a neural associate of reward learning deficits in MDD. However, this observed heterogeneity between studies may indicate that that the findings may not be demonstrating blunting in the same striatal process.

In terms of striatal responses to negative feedback during learning, some studies reported normal VS sensitivity to negative feedback on learning tasks (Hall et al., 2014; Robinson et al., 2012); conversely, Ubl and colleagues found enhanced VS prediction error signalling for unexpected losses on a probabilistic reward task (Ubl, Kuehner, Kirsch, Ruttorf, Diener, et al., 2015b). Antidepressant use may have caused this heterogeneity, as antidepressants normalize behavioural sensitivity to negative feedback (Herzallah et al., 2013), and unmedicated MDD patients tend to be hyperresponsive to negative feedback (Eshel & Roiser, 2010; Herzallah et al., 2013). One study, here, using a medicated sample, observed normal striatal sensitivity to negative feedback (Hall et al., 2014), whereas another, using an unmedicated sample, observed hyperresponsive prediction error signalling to negative feedback (Ubl, Kuehner, Kirsch, Ruttorf, Diener, et al., 2015b). However, one identified study also using unmedicated MDD participants did not report any striatal abnormalities on a reversal learning task (Robinson et al., 2012). The two identified studies using unmedicated participants may have differed because the former used an algorithm to calculate prediction errors (Ubl, Kuehner, Kirsch, Ruttorf, Diener, et al., 2015b), whereas the latter did not calculate prediction errors (Robinson et al., 2012); this may have contributed to the latter study not identifying any striatal changes to negative feedback (Robinson et al., 2012). Taken together, striatal hypersensitivity to negative feedback during learning may be a neural underpinning of only unmedicated MDD, but it could also be argued that striatal hypersensitivity to losses in MDD could be related specifically to hyperactive prediction error processing for losses during reward learning.

In terms of frontal areas, both vmPFC hypoactivation in response to negative feedback (Hall et al., 2014), and OFC hypoactivation during response to positive feedback (Rothkirch et al., 2017; Segarra et al., 2016) has been reported. This suggests a consistent pattern of frontal hypoactivity in the OFC and vmPFC regions alongside reward learning deficits in MDD, especially since two of the three studies identified higher anhedonia scores in MDD participants versus controls using the SHAPS (Rothkirch et al., 2017; Segarra et al., 2016). Interestingly, these patterns were observed across different task types, including reversal learning (Hall et al., 2014), the slot machine task (Segarra et al., 2016), and an instrumental reward learning task (Rothkirch et al., 2017), and assessing responses to positive (Rothkirch et al., 2017; Segarra et al., 2016) versus negative feedback (Hall et al., 2014). In future, further investigations of frontal abnormalities in response to positive and negative feedback during reward learning would be useful to enhance support for this conclusion.

In terms of cingulate abnormalities, a recent finding indicated blunted ACC prediction error signalling for unexpected rewards (Ubl, Kuehner, Kirsch, Ruttorf, Diener, et al., 2015b). Although no study has yet replicated this finding, we believe it is relatively robust, as they calculated prediction errors, and the result is consistent with the lack of inverse relationship between reward expectancy and striatal prediction error signalling, mentioned above (Greenberg et al., 2015). Furthermore, blunted ACC prediction error signalling underpinning reward learning deficits in MDD concurs with the role of this area in reward processing, as the ACC codes prediction error signals for unexpected rewards (Hayden, Heilbronner, Pearson, & Platt, 2011), to guide reward-related behaviour based on previous reinforcement (Der-Avakian & Markou, 2012; Kennerley, Walton, Behrens, Buckley, & Rushworth, 2006; Rushworth & Behrens, 2008).

### Summary of findings

Here, a tentative integration is provided based on the most robust neuroimaging findings within each component of anhedonia, in order to elucidate findings from the literature.

#### Reward liking (consummatory phase)

Striatal hypoactivation was observed in the VS, NAc, caudate, putamen and right anterior insula, which could be associated with abnormal opioid signalling. Frontal hyperactivation was observed in areas including the vmPFC, mPFC, and dlPFC, as well as the frontal hypoactivation in the OFC.

#### Reward wanting (anticipatory phase)

Striatal hypoactivation was observed in the VS/NAc, caudate, and putamen, which could be associated with abnormal dopamine signalling. Frontal hypoactivation was observed in the OFC. There was a trend towards hyperactivation in other frontal areas, including the middle frontal gyrus, mPFC, and dlPFC, as well as a trend towards ACC hyperactivation.

#### Reward wanting (incentive motivation)

Striatal hypoactivation was observed, which could be associated with abnormal dopamine signalling. Abnormal activation in the OFC and ACC was also observed, but requires further replication to be comprehensively understood

#### Reward learning

Blunted striatal prediction error signalling was observed in the VS as well as striatal hypoactivity for unexpected rewards, which could be associated with abnormal striatal dopamine signalling. Frontal hypoactivation in the vmPFC and OFC was observed in response to feedback in reward learning, as well as blunted ACC prediction errors in response to unexpected rewards.

### Common and dissociable neural underpinnings

There are common frontostriatal abnormalities underpinning deficits across the three subtypes of anhedonia in MDD. However, on closer inspection, these common neural patterns are partially dissociable across the subtypes, in line with previous reviews suggesting the three reward processing subtypes are partially dissociable in the healthy brain (Rømer Thomsen et al., 2015), and deficits across the distinguishable components of anhedonia may have partially dissociable neural bases (Admon & Pizzagalli, 2015; Husain & Roiser, 2018). Indeed, a recent review suggests individuals with anhedonia could have different, particular combinations of dissociable neural underpinnings contributing to their anhedonic phenotype (Husain & Roiser, 2018).

Striatal hypoactivation is common across the components of anhedonia in MDD, but may be associated with distinct neurotransmitter systems. For example, striatal hypoactivation for reward liking deficits may be associated with dysfunctional opioid signalling, as striatal opioids code our core liking responses (Berridge & Kringelbach, 2008, 2013; Kelley et al., 2002), and there are abnormalities in opioid signalling both in MDD (Kennedy et al., 2006), and in suicide victims (Zalsman et al., 2005). In contrast, striatal hypoactivation for reward wanting deficits (anticipation and reward selection and approach components of anhedonia) may be associated with dysfunctional dopamine signalling, because dopamine in the striatum is involved in both coding reward expectancy (Abler et al., 2006), and in driving behaviour towards the highest reward outcome (Bardgett et al., 2009; Denk et al., 2005; Salamone et al., 2007; Salamone et al., 2012). Finally, for reward learning, studies have calculated blunted prediction error signalling in the striatum (Gradin et al., 2011; Greenberg et al., 2015; Kumar et al., 2008), and this may be associated with dysfunctional midbrain dopamine signalling, as midbrain dopamine neurons projecting to the striatum code prediction errors (Abler et al., 2006; Bayer & Glimcher, 2005; Schultz, 1997, 1998), to guide reward-related behaviour (Glimcher, 2011). Additionally, there are abnormalities in dopamine signalling in MDD (Dailly et al., 2004; Dunlop & Nemeroff, 2007; Nestler & Carlezon, 2006), and these striatal abnormalities have been observed during the reward processing MID task in MDD (Hamilton et al., 2018). Therefore, striatal hypoactivation associated with deficits in reward liking, wanting and learning, potentially has differential associations with abnormalities in striatal opioid and dopamine function, making it a partially dissociable neural underpinning of the components of anhedonia.

The results of this review suggest reward liking and wanting deficits in MDD are associated with frontal hyperactivation, in areas such as the mPFC and dlPFC, whereas OFC hypoactivation is observed across all three subtypes. Different OFC regions have dissociable functions in reward processing, so OFC hypoactivation itself may be also a partially dissociable neural underpinning of the components of anhedonia. Indeed, midanterior OFC regions integrate reward valence with state for conscious hedonic experience (Berridge & Kringelbach, 2008), so midanterior dysfunction may underpin reward liking and anticipation deficits. However, medial OFC regions are involved in reward monitoring (Berridge & Kringelbach, 2008), allowing the OFC to hold and update stimulus-reinforcement representations (O’Doherty, 2004; Pizzagalli, 2014), so medial OFC dysfunction may conversely be associated with reward learning deficits.

### Limitations

#### Reward processing tasks

A strength of the variety of tasks used in the identified studies is that they vary in the type of reward stimuli presented to assess hedonic response, how participants gain these rewards, and the type of learning feedback, so similar findings across tasks within a reward processing subtype appear robust.

However, similar reward processing tasks have been used across neuroimaging studies investigating reward liking, wanting, and learning. This overlap in task use across the subtypes of anhedonia is a limitation of the current evidence base, because it makes it unclear which partially dissociable subtype of reward processing the neural abnormalities are associated with.

Firstly, the delivery phases of the MID and card guessing task have been used to assess neural abnormalities associated with reward liking, and the anticipation phases of these tasks have also been used to assess the neural abnormalities associated with reward wanting. In these tasks, the reward delivery and anticipation phases are temporally close together, so the studies may have experienced interference between the two reward processes in their neuroimaging analysis, meaning it would be more difficult to detect the specific neural abnormalities underpinning these two reward processes. Additionally, dissociability in the neural abnormalities for reward liking and reward wanting is difficult to distinguish.

Secondly, similar reward processing tasks have been used by certain studies, but these studies have interpreted the results as relating to different reward processing subtypes. Indeed, some reward learning studies have assessed neural abnormalities during delivery of unexpected rewards, and interpreted their results in terms of neural reward learning signals in MDD (Segarra et al., 2016), whereas reward liking studies have used similar tasks and the same task phase to assess neural responses to the experience of rewards. The activation abnormalities observed in response to unexpected rewards in these tasks could either represent abnormal prediction error signals, associated with reward learning, or abnormal neural signals for hedonic response to rewards, associated with reward liking (Segarra et al., 2016). Therefore, in future, reward learning studies using learning tasks or calculating prediction errors may be useful for clarifying the separable neural underpinnings of reward learning deficits in MDD.

#### Sample characteristics

Another limitation is that the characteristics of the patient samples vary across all identified studies, in terms of the sample size, age of their populations, whether the MDD patients were taking medication, whether the participants had remitted or current MDD, and the average depression rating scale scores. Furthermore, although the majority of studies used the DSM-IV as their diagnostic criteria, assessed by the structured clinical interview (SCID), some studies assessed DSM-IV diagnosis using clinical assessment rather than the SCID (Gradin et al., 2011; Johnston et al., 2015; Kumar et al., 2008), one study used an ICD-10 diagnosis of MDD (Keedwell et al., 2005), and studies in children and adolescents assessed diagnosis using the K-SADS-PL (Forbes et al., 2006; Forbes et al., 2009; Morgan et al., 2016; Shad et al., 2011).

Another limitation of the sample characteristics is that studies varied as to whether they measured and reported the anhedonia scores of MDD participants, what scales were used to assess anhedonia levels, and how certain scales were scored (e.g., the SHAPS). This variability makes it difficult to ascertain the extent to which certain findings relate specifically to the experience of anhedonia in the MDD participants rather than to MDD in general, and to assess the relationship between severity of anhedonia levels in individuals and the observed neural changes. However, as indicated above, certain findings were strengthened by studies that measured higher anhedonia levels in MDD participants than controls, and, importantly, by studies additionally investigating correlations between anhedonia severity and the observed neural underpinnings.

#### Neuroimaging

There was also heterogeneity in terms of the fMRI analysis methodology. For example, some studies used ROIs analysis, whereas others conducted whole brain analysis and, across the studies using ROIs, heterogeneous brain regions were used. Therefore, the neural activations observed in MDD may have been made heterogeneous by the varying sample and analysis characteristics, thus obscuring the neural abnormalities detected in MDD patients when looking across studies within each subtype of anhedonia.

Furthermore, although neuroimaging is an essential tool for understanding the activation patterns associated with the three subtypes of anhedonia, a limitation of the neuroimaging evidence base is that only one identified study simultaneously assayed neurotransmitter function using PET (Hamilton et al., 2018). As mentioned above, striatal hypoactivation across the three subtypes of anhedonia may have partially dissociable associations with neurotransmitter functions. However, currently, it is difficult to discern whether striatal hypoactivation is a partially dissociable neural underpinning of the three subtypes of anhedonia, based on neurotransmitter dysfunction, because we cannot determine whether the predicted neurotransmitter dysfunction patterns are present. Therefore, further studies using simultaneous fMRI and PET would be useful to improve our understanding of the dissociability of neural abnormalities across the three subtypes of anhedonia.

### Clinical implications

The results of this review, through contributing to enhancing our understanding of the neurobiology of anhedonia in MDD, have future clinical implications in terms of developing treatments better addressing anhedonia and identifying markers of treatment response. Indeed, although two identified studies did not find an association with anhedonia (Arrondo et al., 2015; Connolly et al., 2015), five others did report a relationship between the neural patterns related to reward processing deficits and anhedonia levels in MDD (Epstein et al., 2006; Keedwell et al., 2005; Misaki et al., 2016; Rothkirch et al., 2017; Stringaris et al., 2015; Young et al., 2016) and remitted MDD (Geugies et al., 2019). This tentatively suggests that the observed neurophysiological processes underpinning reward processing do have a relationship with the experience of anhedonia in MDD.

With this in mind, better understanding the neural abnormalities underpinning reward processing deficits in MDD should be able to contribute the development of pharmacotherapies targeting these neural abnormalities and, therefore, anhedonia. For example, new treatments could be developed to remediate striatal hypoactivation, such as drugs modulating dopamine function. Indeed, as mentioned previously, striatal hypoactivation in reward wanting may be associated with dopamine signalling dysfunction, and, furthermore, there are abnormalities in MDD in striatal dopamine function (Dailly et al., 2004; Nestler & Carlezon, 2006), which are associated with anhedonia (Argyropoulos & Nutt, 2013). Preliminary evidence shows that dopamine-targeting drugs such as bupropion (Tomarken, Dichter, Freid, Addington, & Shelton, 2004) and aripiprazole are effective at reducing anhedonia in MDD (Reimherr et al., 2010), and, furthermore, increasing dopamine transmission using amisulpride ameliorates reward-related striatal hypoactivation in MDD (Admon et al., 2017). Opioid function is also abnormal in MDD (Kennedy et al., 2006; Zalsman et al., 2005), and, as mentioned previously, may be associated with striatal hypoactivation in reward liking, but there are only preliminary results thus far suggesting opioid-targeting drugs could have antidepressant properties (Ehrich et al., 2015; Karp et al., 2014). In future, further clinical studies investigating these novel, targeted pharmacotherapies will be useful to assess their efficacy for improving anhedonia and remediating its neural underpinnings.

Dopamine-targeting and opioid-targeting drugs, however, are not the only options for new pharmacotherapies targeting the neural abnormalities of anhedonia. Indeed, various abnormalities in the frontostriatal reward processing network are associated with anhedonia, but are also associated with inflammatory activation (Swardfager, Rosenblat, Benlamri, & McIntyre, 2016). For example, endotoxin-induced inflammation is associated with striatal hypoactivation for rewards (Eisenberger et al., 2010), and increased C-reactive protein levels are associated with decreased VS to vmPFC connectivity, which in turn correlates with anhedonia (Felger et al., 2016). Therefore, an alternative treatment targeting the neural abnormalities associated with anhedonia could be anti-inflammatory drugs (Swardfager et al., 2016). So far, evidence relating anti-inflammatory treatments to anhedonia is sparse, but the anti-inflammatory antibiotic minocycline has been shown to attenuate lipopolysaccharide-induced inflammatory activation, and thus prevent anhedonic-like behaviour in mice (Henry et al., 2008). Therefore, future studies could build on this result to determine whether anti-inflammatory treatments could be developed to remediate both anhedonia and its neural underpinnings.

Importantly, the presence of partially dissociable neural underpinnings across the components of anhedonia could indicate the potential for variability across individuals with an anhedonic phenotype in terms of their particular combination of underlying, disrupted neurobiological mechanisms (Husain & Roiser, 2018). The treatment options mentioned above may be useful for addressing these disrupted mechanisms, but it would be useful to identify the individual combination of disrupted mechanisms in order to develop personalized interventions for anhedonia (Husain & Roiser, 2018), such as the patient’s individual neurotransmitter alterations affecting striatal regions and/or changes in activity in different frontal regions.

Finally, enhancing our neurobiological models of reward processing deficits in depression will aid in treatment development, by allowing the assessment of how new treatments impact on the frontostriatal abnormalities associated with reward processing deficits and, therefore, anhedonia. For example, behavioural activation therapy results in functional changes in frontostriatal network activity during the WoF task (Dichter et al., 2009), suggesting this therapy modality may be particularly useful for patients with anhedonia, through altering neural activity during reward processing. This type of study can also be attempted in other treatment modalities, such as dopamine-targeting and opioid-targeting drugs and anti-inflammatory drugs, to determine whether targeted treatments could be used to remediate the neural abnormalities underpinning the components of reward processing deficits in anhedonia. For example, a proof-of-mechanism trial observed that 8-week use of a κ-opioid receptor agonist increased VS activation during reward anticipation versus placebo in patients with anhedonia and a mood or anxiety disorder (Krystal et al., 2020), indicating this treatment does have specific effects on the functioning of reward circuitry in these patients. Indeed, using biomarkers of neural effects which have potential therapeutic benefit will be very useful in future development of pharmacological and psychological treatments for anhedonia as important outcome measures for proof of mechanism studies such as this (Krystal et al., 2018), as this reduces the risk of failure in later trials by showing whether therapeutic effects are due to certain neural reward circuitry alterations rather than other factors (Krystal et al., 2018).

## Conclusions

In conclusion, this systematic review summarized both common and dissociable neural underpinnings for deficits across the three subtypes of anhedonia in MDD: reward liking, reward wanting, and reward learning. For reward liking and reward wanting, studies showed consistent striatal hypoactivation, alongside hypoactivation and hyperactivation across dissociable frontal regions. Conversely, for reward learning, studies showed blunted frontostriatal sensitivity in response to positive feedback, but no neural abnormalities for negative feedback. These findings suggest the importance of studying anhedonia not only as a clinical manifestation but also as a neurobiological mechanism underlying depressive disorder and other psychiatric conditions. Developing more comprehensive neurobiological models of anhedonia will have clinical implications, as, firstly, it will aid the development of novel pharmacotherapies targeting the neural abnormalities underpinning anhedonia, and, secondly, it will allow us to determine whether targeted treatments do indeed remediate address these neural abnormalities better than current antidepressant treatments.
